# Porous V_2_O_5_/RGO/CNT hierarchical architecture as a cathode material: Emphasis on the contribution of surface lithium storage

**DOI:** 10.1038/srep31275

**Published:** 2016-08-11

**Authors:** Kowsalya Palanisamy, Ji Hyun Um, Mihee Jeong, Won-Sub Yoon

**Affiliations:** 1 Department of Energy Science, Sungkyunkwan University, Suwon, 440-746, South Korea; 2 Integrated Energy Center for Fostering Global Creative Researcher, Sungkyunkwan University, Suwon 440-746, South Korea

## Abstract

A three dimensional vanadium pentoxide/reduced graphene oxide/carbon nanotube (3D V_2_O_5_/RGO/CNT) composite is synthesized by microwave-assisted hydrothermal method. The combination of 2D RGO and 1D CNT establishes continuous 3D conductive network, and most notably, the 1D CNT is designed to form hierarchically porous structure by penetrating into V_2_O_5_ microsphere assembly constituted of numerous V_2_O_5_ nanoparticles. The highly porous V_2_O_5_ microsphere enhances electrolyte contact and shortens Li^+^ diffusion path as a consequence of its developed surface area and mesoporosity. The successive phase transformations of 3D V_2_O_5_/RGO/CNT from α-phase to ε-, δ-, γ-, and ω-phase and its structural reversibility upon Li^+^ intercalation/de-intercalation are investigated by *in situ* XRD analysis, and the electronic and local structure reversibility around vanadium atom in 3D V_2_O_5_/RGO/CNT is observed by *in situ* XANES analysis. The 3D V_2_O_5_/RGO/CNT achieves a high capacity of 220 mAh g^−1^ at 1 C after 80 cycles and an excellent rate capability of 100 mAh g^−1^ even at a considerably high rate of 20 C. The porous 3D V_2_O_5_/RGO/CNT structure not only provides facile Li^+^ diffusion into bulk but contributes to surface Li^+^ storage as well, which enables the design of 3D V_2_O_5_/RGO/CNT composite to become a promising cathode architecture for high performance LIBs.

Lithium ion batteries (LIBs) have gained great attention in scientific and industrial fields due to their high working voltage, high capacity, and long cycling life, and its applications are being expanded to portable electronic devices, electric vehicles, and power grids^1^. For the extensive applications of LIBs, the desire for high energy has been increased, which necessitates the development of cathode material with a higher capacity and a higher working voltage^2^. Until now, the most-studied cathode materials deliver capacities lower than 200 mAh g^−1^ including the commercialized cathode materials such as LiCoO_2_ (140 mAh g^−1^), LiFePO_4_ (170 mAh g^−1^), and LiMn_2_O_4_ (148 mAh g^−1^)^1^,^3^,^4^. In contrast, vanadium pentoxide (V_2_O_5_) has a relatively high theoretical capacity of 294 mAh g^−1^ based on 2 Li^+^ intercalation/de-intercalation per unit formula in the voltage range of 4.0–2.0 V (*vs.* Li/Li^+^ ), and its layered crystal structure makes it a host for reversible Li^+^ intercalation/de-intercalation^5^. During the last 40 years since the first report on the reversible intercalation of Li^+^ in V_2_O_5_^6^, various nanostructured V_2_O_5_ have been studied as a promising cathode material, but sluggish Li^+^ diffusion coefficient (10^−12^ cm^2^ s^−1^) and low electrical conductivity (10^−4^~10^−5^ S cm^−1^) have hindered its practical application^7^.

Constructing nanometer-scale architecture with conductive carbon-based materials is one of the strategies for enhancing the energy and power densities by shortening Li^+^ transport path and increasing electrical conductivity at the same time, and many conductive materials such as mesoporous carbon^8^, carbon nanotube (CNT)^9^, graphene sheet^10^, graphene foam^11^, and conductive polymer^12^,^13^ have been investigated. Among them, graphene sheets with a two-dimensional (2D) structure have the advantage of being used as a substrate for embedded metal oxide due to mechanical strength, chemical stability, extraordinary electrical conductivity of 10^3^~10^4^ S m^−1^, and ultrahigh theoretical surface area of 2630 m^2^ g^−110^. Studies of V_2_O_5_ hybridized with 2D graphene sheets have been extensively accomplished by controlling the dimensionality of V_2_O_5_ such as zero-dimensional (0D) nanoparticles^14^ or quantum dots^15^, one-dimensional (1D) ribbons^16^ or nanowires^17^, 2D nanosheets^18^, and three-dimensional (3D) aerogel^19^ or hydrogel^20^, which significantly improves the electrochemical performance. However, especially for the 0D V_2_O_5_/graphene nanocomposite architecture, we believe that considerable room for improvement in the electrode structure is left because nanomaterials are often self-aggregated or dissolved during cycling due to its high surface energy^21^. Furthermore, when a hierarchical assembly structure is designed to solve the above problems, a strategy for increasing the surface area can be possible by the introduction of materials with another dimensionality. By employing the 1D CNT, metal oxide/graphene/CNT ternary composites have been reported^22^,^23^, and the role of CNT in the composites is generally expected to avoid the restacking of graphene sheets as a pillar and to enhance the electron transport by constructing 3D electrical conductive networks^24^. However, the increase in surface area of the ternary composite by the introduction of CNT is yet insignificant^25^,^26^ compared to metal oxide/graphene binary composite, or otherwise the surface area rather decreases with the addition of CNT^27^. Beyond the typical role of CNT pillar, a hierarchically porous assembly structure can be suggested to dramatically increase the surface area by using the CNT which is designed to penetrate into the assembly structure. Therefore, it is necessary and meaningful to investigate the addition effect of CNT on the surface area in the ternary composite for constructing a hierarchical structure.

Herein, we demonstrate a porous 3D V_2_O_5_/graphene/CNT ternary composite by using 2D reduced graphene oxide (RGO) and 1D CNT as conductive network through microwave-assisted hydrothermal (MAH) method (hereafter, referred to as 3D V_2_O_5_/RGO/CNT). In hierarchically porous assembly structure, the V_2_O_5_ microsphere is constituted by numerous V_2_O_5_ nanoparticles and simultaneously penetrated by CNT on the surface of RGO. In particular, the penetrating CNT is designed to increase surface area, which enhances electrolyte contact and Li^+^ diffusivity. The phase transformations upon Li^+^ intercalation/de-intercalation and the variation of electronic and local structure around vanadium atom in the 3D V_2_O_5_/RGO/CNT composite are investigated by *in situ* XRD and *in situ* XANES analyses, respectively. This hierarchically porous structure demonstrates a high reversible capacity and excellent rate capability with stable capacity retention, and the design of 3D V_2_O_5_/RGO/CNT composite by taking the advantage of porous structure is favorable to the Li^+^ diffusion into bulk and the capacitive Li^+^ storage on surface, which can be a rational design to enhance the bulk utilization and additional surface storage.

## Results and Discussion

### Porous V_2_O_5_/RGO/CNT hierarchical architecture

**Figure 1:**
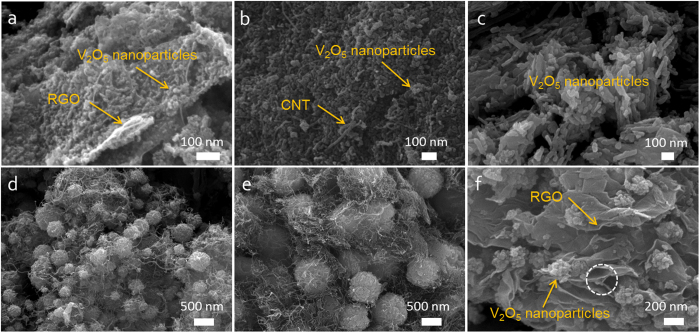
FE-SEM images of (**a**) V_2_O_5_ with RGO, (**b**) V_2_O_5_ with CNT, (**c**) only V_2_O_5_ nanoparticles, (**d**) and (**e**) 3D V_2_O_5_/RGO/CNT composites with the controlled ratio of RGO to CNT at 1:2 and 2:1, respectively, and (**f**) 2D V_2_O_5_/RGO composite.

One-step MAH method which is an energy effective dielectric heating system for producing high quality nanomaterials under fast kinetics of crystallization^28^ was applied to obtain the 3D V_2_O_5_/RGO/CNT composite. Acid treated o-CNTs were well dispersed in amphiphilic GO solution through π-π interaction, and then reduction of V_2_O_5_ precursor had occurred in the mixture of GO and o-CNT with the reducing agent of ascorbic acid by forming V_x_O_y_/RGO/CNT ternary composite. Through the oxidation process, a 3D V_2_O_5_/RGO/CNT ternary microsphere was obtained and applied to cathode material. To confirm the individual role of RGO and CNT in the formation of hierarchical microsphere, V_2_O_5_ with only RGO and only CNT were prepared separately. The SEM images of Fig. 1a,b show the morphology of only V_2_O_5_/RGO and only V_2_O_5_/CNT, respectively. Instead of the V_2_O_5_ nanoparticle assembly, the V_2_O_5_ nanoparticles are totally dispersed onto the surface of 2D RGO (Fig. 1a) or agglomerated each other (Fig. 1b) in the similar way when only V_2_O_5_ nanoparticles without RGO or CNT undergo the MAH process (Fig. 1c). However, when the RGO and CNT were added together, the V_2_O_5_ assembly was obtained as shown in Fig. 1d,e. From the totally different morphologies even through the essentially same synthesis process, the RGO/CNT structure can be regarded as a critical role in forming the V_2_O_5_ microsphere. A local interspace confined by RGO and CNT can be a reason for the assembly structure because the V_2_O_5_ nanoparticles in the local interspace grow into microsphere via an Ostwald ripening process^29^. Figure 1d,e present the morphology of 3D V_2_O_5_/RGO/CNT composite with the ratio of RGO to CNT at 1:2 and 2:1, respectively. Compared to Fig. 1e, the smaller V_2_O_5_ assemblies are observed in Fig. 1d at the same magnification, and the particle size distribution of V_2_O_5_ assemblies is not uniform. Moreover, the spheres are less covered with RGO and CNT, as a result, the ratio of RGO:CNT was controlled at 2:1 for subsequent experiments. Based on the local interspace confining the V_2_O_5_ nanoparticles and reconstructing them, the amount of RGO was increased twice to confirm the possibility for forming the V_2_O_5_ microsphere without the addition of CNT. Only 2D RGOs without CNT can build the confined structure on the assumption that the increased concentration of RGO has more opportunity to obtain a standing RGO between parallel RGOs and thereby makes the local interspace. As shown in Fig. 1f, the V_2_O_5_ assembly structure is developed, and irregular V_2_O_5_ nanoparticle aggregates over at least 100 nm are randomly anchored on the surface of RGO, in contrast to the only V_2_O_5_/RGO at the low concentration of RGO (Fig. 1a). Although there is an interconnecting RGO between two RGOs as marked with a dotted circle in Fig. 1f, most RGOs seem to be stacked, therefore, it is referred to as 2D V_2_O_5_/RGO and regarded as a control group for investigating the effect of morphological difference between RGO and CNT on V_2_O_5_ microsphere structure and its electrochemical properties.

**Figure 2:**
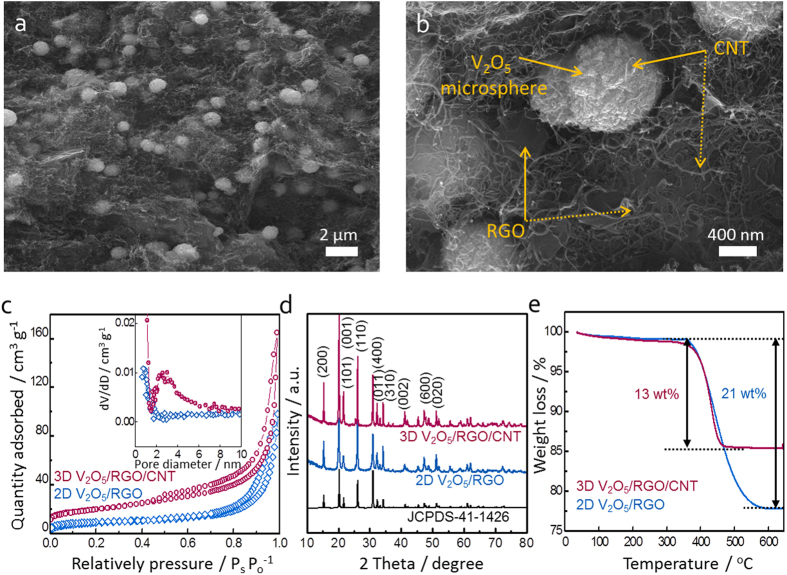
(**a,b**) FE-SEM images of 3D V_2_O_5_/RGO/CNT composite at low and high magnifications, respectively; (**c**) N_2_ adsorption/desorption isotherms of 3D V_2_O_5_/RGO/CNT and 2D V_2_O_5_/RGO composites and their pore size distributions (inset); (**d**) X-ray diffraction patterns of the hybrid composites with an orthorhombic V_2_O_5_ structure (JCPDS-41-1426); (**e**) TGA profiles of the hybrid composites.

The SEM image of Fig. 2a presents that the V_2_O_5_ nanoparticle aggregates below 1 μm are randomly dispersed in the architecture of 3D V_2_O_5_/RGO/CNT. At higher magnification in Fig. 2b, CNT touching the surface of V_2_O_5_ microsphere is observed as indicated by solid arrow, and most notably, certain CNT among the entangled CNTs seems to penetrate the RGO as indicated by dotted arrow. In respect of RGO, the porous RGO interpenetrated by CNTs (dotted arrow) as well as the smooth RGO (solid arrow) are observed, in contrast to the only smooth surface of RGO in 2D V_2_O_5_/RGO, which can indicate the 3D conducting scaffold composed of RGO and CNT in the ternary composite. To investigate the texture properties of composite, N_2_ adsorption/desorption measurements of 3D V_2_O_5_/RGO/CNT and 2D V_2_O_5_/RGO composites were performed as shown in Fig. 2c. The isotherm profile of 3D V_2_O_5_/RGO/CNT composite corresponds to type IV with a hysteresis loop around 0.5–0.7 P/P_0_, suggesting a mesoporous structure, whereas that of 2D V_2_O_5_/RGO is categorized as type II, indicating a negligible development of mesopores (2~50 nm)^30^. The specific surface area and pore volume of 3D V_2_O_5_/RGO/CNT composite are calculated as 70 m^2^ g^−1^ and 0.25 cm^3^ g^−1^, respectively which are much higher than that of 2D V_2_O_5_/RGO (32 m^2^ g^−1^ and 0.15 cm^3^ g^−1^, respectively), indicating that the introduction of 1D CNT remarkably enhances the specific surface area and porosity. For comparison, specific surface area and pore volume of V_2_O_5_ nanoparticles with only RGO and only CNT are 23 m^2^ g^−1^ and 0.10 cm^3^ g^−1^ and 17 m^2^ g^−1^ and 0.08 cm^3^ g^−1^, respectively. The pore size distributions of 3D V_2_O_5_/RGO/CNT and 2D V_2_O_5_/RGO composites are shown in the inset of Fig. 2c. The mesopores ranging from 2 to 10 nm are obviously developed in the ternary composite, whereas the amount of mesopores in 2D V_2_O_5_/RGO is negligible. The mesopores within 2~10 nm in 3D V_2_O_5_/RGO/CNT composite can be resulted from the interspaces of constituent particles, which means the increased gaps between the V_2_O_5_ nanoparticles and concurrently emphasizes the role of CNT in the composite for increasing the mesoporosity and consequently specific surface area. According to the XRD patterns in Fig. 2d, all diffraction peaks of 3D V_2_O_5_/RGO/CNT and 2D V_2_O_5_/RGO composites are indexed to pure orthorhombic crystalline phase of V_2_O_5_ (JCPDS-41-1426)^31^. No characteristic RGO and/or CNT peaks in the hybrid composites are observed because the first main peak of RGO and/or CNT overlaps with (110) reflection of V_2_O_5_ at around 27°^ ^32. To determine the carbon content in hybrid composites, TGA was conducted in air. As shown in Fig. 2e, a single step of weight loss between 300–650 °C is observed in both samples, corresponding to the oxidation of the carbon^16^. Excluding physically adsorbed water (below 200 °C), the carbon contents of 3D V_2_O_5_/RGO/CNT and 2D V_2_O_5_/RGO composites are evaluated to be about 13 and 21 wt%, respectively.

**Figure 3:**
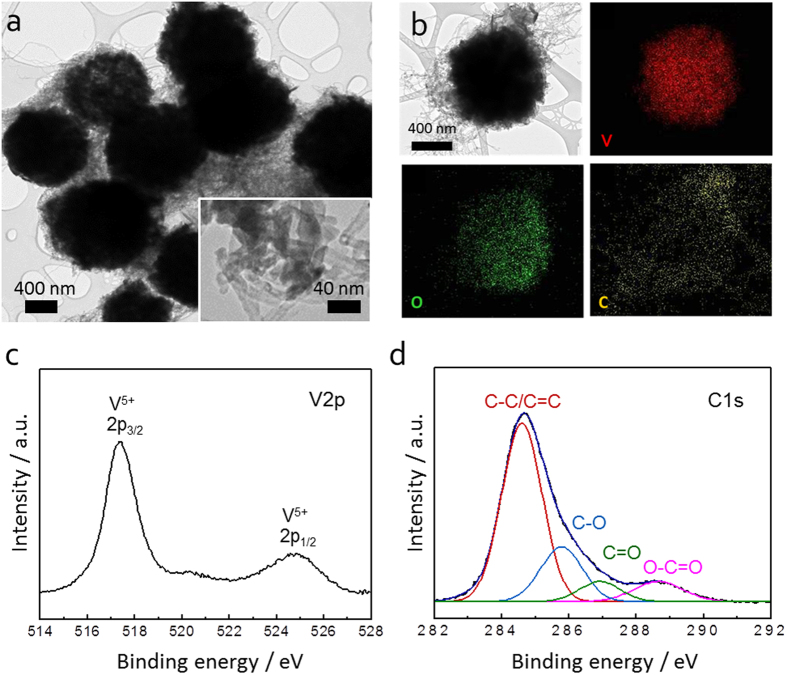
(**a**) HR-TEM image of 3D V_2_O_5_/RGO/CNT composite at low magnification (Inset shows the V_2_O_5_ assembly penetrated by CNT at high magnification); (**b**) elemental mapping images of 3D V_2_O_5_/RGO/CNT composite with vanadium (red), oxygen (green), and carbon (yellow); (**c,d**) XPS spectrum of 3D V_2_O_5_/RGO/CNT composite at V2p and at C1s, respectively.

The hierarchical structure of 3D V_2_O_5_/RGO/CNT composite was further examined by using HR-TEM and EDX mapping images. As shown in Fig. 3a, the V_2_O_5_ microspheres about 1 μm are embedded in RGO/CNT matrix. The inset at higher magnification exhibits the CNTs penetrating into the V_2_O_5_ assembly, which well describes the porous nature of hierarchical structure. Moreover, the element distributions of V, O, and C are homogeneous in a whole microsphere (Fig. 3b). XPS was carried out to analyze the chemical state of vanadium and the reduction extent of GO in the ternary composite. For the V2p spectrum (Fig. 3c), the binding energy separation between V2p_1/2_ (525.1 eV) and V2p_3/2_ (517.4 eV) is ~7.7 eV, which corresponds with +5 oxidation state of V_2_O_5_^33^. The peak fitting of C1s spectra in Fig. 3d shows the four main peaks at 284.7, 286.8, 288.3, and 288.3 eV, assigned to C-C/C=C (aromatic ring), C-O (hydroxyl or epoxy group), C=O (carbonyl functional group), and O-C=O (carboxyl group), respectively. Although the C1s spectrum is mixed with RGO and CNT, the highest intensity in the C-C/C=C peak at 284.4 eV suggests the reduction of oxygen functional groups within RGO and the effective restoration of sp^2^ carbon network^34^.

### *In situ* XRD and *in situ* XANES analyses of 3D V_2_O_5_/RGO/CNT upon Li^+^ intercalation/de-intercalation

**Figure 4:**
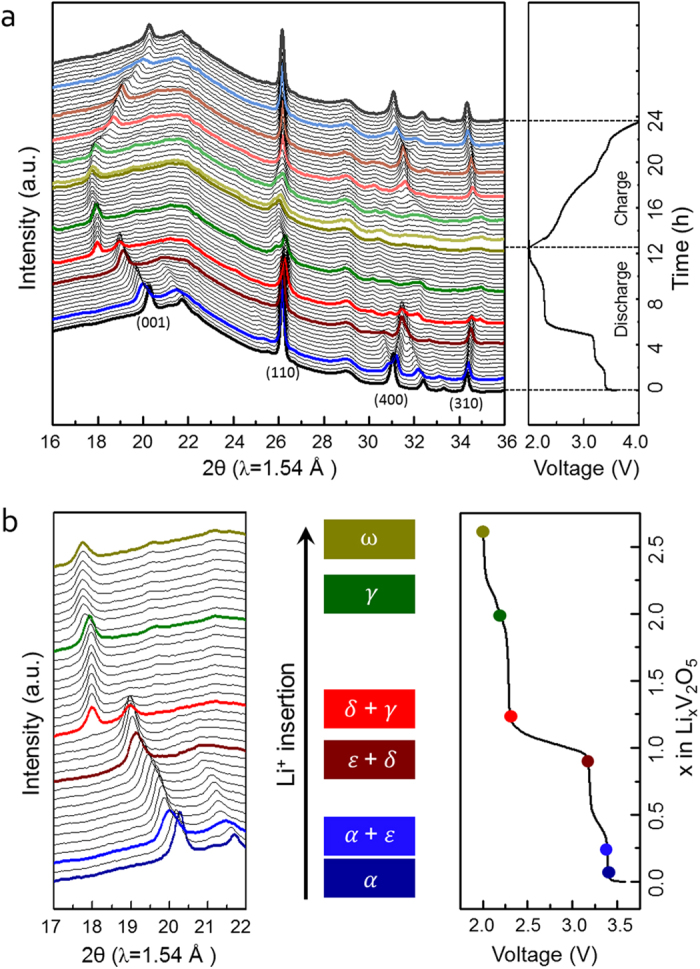
(**a**) *In situ* XRD patterns of 3D V_2_O_5_/RGO/CNT composite during the first discharge and charge processes in the voltage range of 4.0–2.0 V at 0.1 C; (**b**) selected 2θ range during the first discharge process with the voltage profile on the side of XRD data for comparison.

The structural changes of 3D V_2_O_5_/RGO/CNT composite during discharge and charge processes in the voltage range of 4.0–2.0 V at 0.1 C were investigated by *in situ* XRD analysis (Fig. 4). In Fig. 4a, the reflections (i.e. (001), (110), (400), and (310)) continuously shift upon Li^+^ intercalation/de-intercalation, and various phase transitions corresponding to the multi-step process that can be divided into four distinct stages in each discharge and charge voltage profile are observed. The position of all reflections after charge process is nearly the same position at pristine state as indicated by black lines, which reveals a structural reversibility upon Li^+^ intercalation/de-intercalation^35^,^36^. There are numerous metastable phases of Li_*x*_V_2_O_5_ under chemical or electrochemical Li^+^ intercalation into V_2_O_5_ at room temperature^37^,^38^. When the (001) reflection is focused to examine the phase evolutions in the layered V_2_O_5_ structure as a function of Li^+^ composition in the Li_*x*_V_2_O_5_ (Fig. 4b), V_2_O_5_ firstly transforms into α-Li_*x*_V_2_O_5_ in the composition range of *x* < 0.1 and then coexists with ε-Li_*x*_V_2_O_5_ within the composition of *x* = 0.26. This accords with the phase diagram of the Li_x_V_2_O_5_ system at room temperature, indicating that ε-phase exists solely in the composition range of 0.35 < *x* < 0.8^37^. The ε-Li_*x*_V_2_O_5_ accompanies δ-Li_*x*_V_2_O_5_ until the composition of *x* = 0.93, and then the δ-phase is developed into the main structure as further Li^+^ intercalated. At the composition of *x* = 1.27, γ-Li_*x*_V_2_O_5_ appears along with the δ-phase at lower angle and develops until the composition of *x* = 1.93. After that, the γ-phase changes into ω-Li_*x*_V_2_O_5_ and maintains the ω-phase until the end of discharge by uptake of *x* = 2.65. These successive phase transformations from α-phase to ε-, δ-, γ-, and ω-phase are in accord with the general progression of Li^+^ content in the Li_*x*_V_2_O_5_ phases and the four plateaus at 3.4, 3.19, 2.28, and 2.01 V in the first discharge process^37^,^38^,^39^. In addition, the (001) reflection shifts toward lower angles as Li^+^ intercalated, which reflects the increase in the interlayer *d* spacing of V_2_O_5_ layers by uptake of Li^+^, and the intensity of (001) reflection is decreased at the final ω-phase compared to that of α-phase, which indicates the reduction in structural order upon Li^+^ intercalation^35^,^36^. More importantly, however, the lower shift returns to nearly the same position at pristine state, and the decreased intensity recovers a respectable amount of pristine state as shown in Fig. 4a, which confirms the structural reversibility of V_2_O_5_ in the ternary composite upon Li^+^ intercalation/de-intercalation.

**Figure 5:**
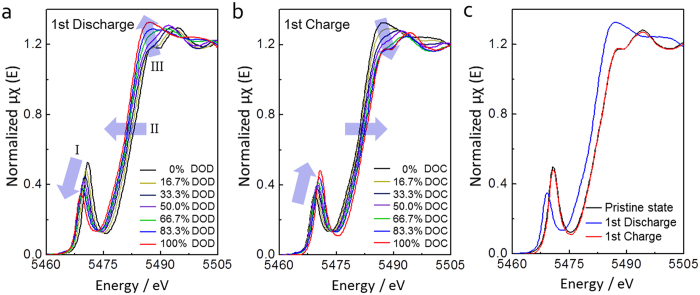
The vanadium K-edge *in situ* XANES spectra of 3D V_2_O_5_/RGO/CNT composite during the first (**a**) discharge and (**b**) charge processes, and (**c**) comparison of the vanadium K-edge XANES spectra at the pristine, 100% discharged, and 100% charged states (DOD and DOC is the depth of discharge and charge, respectively).

Furthermore, to confirm the changes in oxidation state and chemical environment around the vanadium atom in V_2_O_5_ crystals, *in situ* XANES analysis of the 3D V_2_O_5_/RGO/CNT composite was conducted (Fig. 5) during the first discharge and charge processes at 0.1 C. The vanadium K-edge XANES spectra can be divided into the three features, pre-edge, K-edge, and edge resonance as marked with I, II, and III, respectively in Fig. 5a. During the first discharge, the K-edge continuously shifts to the lower energy value as a function of Li^+^ composition in the Li_*x*_V_2_O_5_. This shift represents the decrease in the average oxidation state of vanadium, which corresponds to the increasing amounts of intercalated Li^+ ^40,41. The pre-edge peak decreases in intensity and shifts toward lower energy value as Li^+^ intercalated. The crystalline V_2_O_5_ is composed of VO_5_ square pyramids with highly distorted environment^42^. The intensity of pre-edge peak is considerably sensitive to the alteration of local geometric symmetry and directly correlated with the distortion of VO_5_ square pyramid^40^,^41^. An increase in the intensity of pre-edge peak indicates a decrease in vanadyl (V = O) distance^43^, which increases a degree of distortion of local structure and decreases the local symmetry of structure within the VO_5_ square pyramid^40^,^41^. Therefore, the decreased intensity of pre-edge peak during discharge suggests that the local structure of vanadium at the 100% discharged state has higher degree of local geometric symmetry compared to the pristine state. In accord with the K-edge, the pre-edge peak and edge resonance positions are also seen to shift toward lower energy value as Li^+^ intercalated. The intensity of edge resonance is consistent with the symmetry of basal oxygen configuration around the vanadium site^40^,^41^. As discussed above, the linear decrease in intensity of pre-edge peak represents a reduction of the distortion within VO_5_ square pyramid, and the resultant increase of the basal oxygen configuration around the vanadium site is also observed by an increase in the magnitude of edge resonance as Li^+^ intercalated^41^. All the features observed in the XANES spectra during charge process appear on the contrary to discharge process as indicated by blue arrows in Fig. 5b. As shown in Fig. 5c, the oxidation state of vanadium after the first cycle is extremely close to that of the pristine state, which is based on the overlapped K-edge lines between the pristine and 100% charged states. In addition, after the first cycle, the degree of distortion of the VO_5_ square pyramid decreases, and the symmetry of basal oxygen configuration around the vanadium site slightly increases compared to the pristine state, which is based on the lowered and the slightly raised intensity of the pre-edge and edge resonance at the 100% charged state, respectively. These two XANES results through comparison of the pristine and 100% charged states reveal the electronic and local structure reversibility in the 3D V_2_O_5_/RGO/CNT composite upon Li^+^ intercalation/de-intercalation, respectively^16^,^40^,^41^.

### Li-ion battery performances of 3D V_2_O_5_/RGO/CNT composite

**Figure 6:**
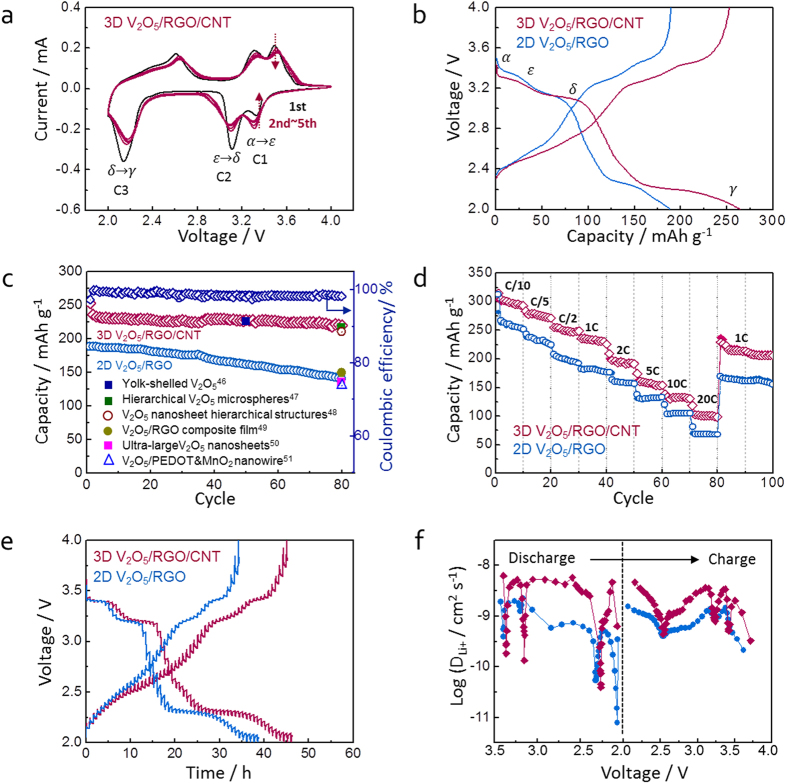
(**a**) Cyclic voltammogram of 3D V_2_O_5_/RGO/CNT composite during the first five cycles at 0.2 mV s^−1^; (**b**) voltage profiles of 3D V_2_O_5_/RGO/CNT and 2D V_2_O_5_/RGO composites at 1 C; (**c**) cycle performance and coulombic efficiency of the hybrid composites at 1 C; (**d**) rate capability of the hybrid composites at various C-rates; (**e**) GITT curves of the hybrid composites as a function of time in the voltage range of 4.0–2.0 V at 0.1 C; (**f**) Li^+^ diffusion coefficients (*D*_Li+_) calculated from GITT profile during the first discharge and charge cycle.

A series of electrochemical measurements were conducted to evaluate the Li^+^ storage properties of 3D V_2_O_5_/RGO/CNT and 2D V_2_O_5_/RGO composites as a cathode material for LIBs. Figure 6a shows the cyclic voltammetry (CV) of 3D V_2_O_5_/RGO/CNT composite during the first five cycles at a scan rate of 0.2 mV s^−1^. In the first CV curve, three cathodic peaks at 3.33, 3.11, and 2.13 V correspond to the structural transformation from the α-phase to the ε-, δ-, and γ-phase, respectively (noted as C1, C2, and C3, respectively, for further explanation in the result section), and three corresponding anodic peaks at 2.61, 3.31, and 3.50 V appear in the following anodic scans. As the cycle repeated, the individual redox peaks are decreased slightly. Except for the absence of final phase transition from γ-phase to ω-phase as further lithiated from γ-phase, the structural transformations accord with the *in situ* XRD results and the previous reports^37^,^39^. Figure 6b presents the voltage profiles of 3D V_2_O_5_/RGO/CNT and 2D V_2_O_5_/RGO composites in the first cycle at 1 C, and C-rate bases on the theoretical capacity of V_2_O_5_ (294 mAh g^−1^). The specific capacity is normalized by the mass of only V_2_O_5_ in the composites because the capacity of RGO and CNT normalized by the carbon content are negligible in the voltage range of 4.0–2.0 V^44^,^45^. In agreement with the CV result, the multiple redox plateaus in the voltage profile show the phase transitions from the α-phase to the ε-, δ-, and finally to the γ-phase, respectively. The first discharge and charge capacities of the 3D V_2_O_5_/RGO/CNT composite are 265 and 253 mAh g^−1^, respectively, and those of the 2D V_2_O_5_/RGO are 190 and 189 mAh g^−1^, respectively, suggesting that more lithium ions with 0.51 Li^+^ are stored in the 3D V_2_O_5_/RGO/CNT than in the 2D V_2_O_5_/RGO composites. The cycle performance (Fig. 6c) presents high capacities and stable capacity retention in the 3D V_2_O_5_/RGO/CNT composite. The capacities of 3D V_2_O_5_/RGO/CNT are 80 mAh g^−1^ higher than the 2D V_2_O_5_/RGO until 80 cycles, and coulombic efficiency of 3D V_2_O_5_/RGO/CNT is 95.5% in the first cycle and then maintains over 99%. The high and stable Li^+^ storage of 3D V_2_O_5_/RGO/CNT composite is superior or fairly comparable to that of recently reported V_2_O_5_-based cathode materials as shown in Fig. 6c 46,47,48,49,50,51. The rate capability of composites was evaluated by increasing the C-rate from 0.1 to 20 C and then returning to 1 C as shown in Fig. 6d. When cycled at 0.1 C, the first charge capacity of 304 mAh g^−1^ decreases to 290 mAh g^−1^ for the subsequent 10 cycles, and the capacity become stable as the C-rate increased. Even at a considerably high rate of 20 C, the 3D V_2_O_5_/RGO/CNT composite delivers a high capacity of 100 mAh g^−1^ with good stability, compared to 68 mAh g^−1^ of 2D V_2_O_5_/RGO. When the C-rate is reduced back to 1 C after 80 cycles, the 3D V_2_O_5_/RGO/CNT composite recovers a respectable amount of the first 1 C step capacity, demonstrating its superior rate capability.

Moreover, GITT was employed to understand kinetics related with the configuration of 3D V_2_O_5_/RGO/CNT and 2D V_2_O_5_/RGO composites. The chemical diffusivities of Li^+^ in hybrid composites were evaluated by using the GITT curve (Fig. 6e) during the first cycle at 0.1 C for an interval of 10 min followed by a rest of 40 min to obtain the steady state equilibrium voltage (*E*_*s*_). The Li^+^ diffusion coefficients (*D*Li^+^ ) of hybrid composites were calculated from the following equation 1 based on Fick’s law^52^


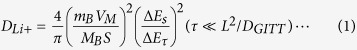


where *m*_*B*_, *V*_*M*_, *M*_*B*_, and *S* are mass, molar volume, molecular weight, and active surface area, respectively; *L* is the characteristic length; Δ*E*_*s*_ is the change in steady-state cell voltage for the step in different potential range; and Δ*E*_*τ*_ is the total change of cell voltage during the current pulse for time τ. As show in Fig. 6f, “W” shape curves of both samples are indicative of the characteristics of Li^+^ intercalation-host materials^53^. The calculated diffusion coefficients of 3D V_2_O_5_/RGO/CNT are higher than those of 2D V_2_O_5_/RGO on the whole process, implying a favorable Li^+^ diffusivity in the ternary composite. However, the *D*_Li+_ of 3D V_2_O_5_/RGO/CNT composite reaches minima values at 3.37, 3.15, and 2.27 V during discharge, corresponding to the plateau voltages of phase transitions in Fig. 6e, and three *D*_Li+_ are lower than 2D V_2_O_5_/RGO composite. It can be explained by the more formation of phase transitions (α-phase to ε-, δ-, and γ-phase, respectively) in the 3D V_2_O_5_/RGO/CNT than in the 2D V_2_O_5_/RGO, which is shown by the fact that every plateau of 3D V_2_O_5_/RGO/CNT is longer than that of 2D V_2_O_5_/RGO^54^.

**Figure 7:**
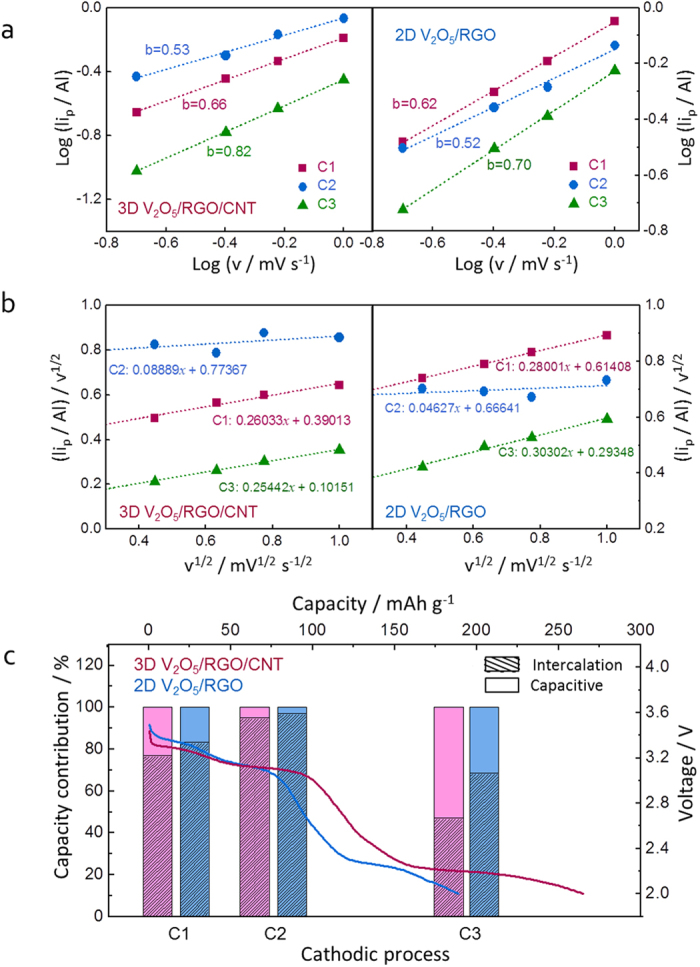
(**a**) Linear relationship of log (|*i*_*p*_|) *vs.* log (ν) in 3D V_2_O_5_/RGO/CNT and 2D V_2_O_5_/RGO composites during cathodic (discharge) sweeps for determining the slope of *b*-value; (**b**) plot of cathodic peak currents depending on scan rate in the hybrid composites for determining the slope of *k*_*1*_ and y-intercept of *k*_*2*_ as the capacitive and diffusion-controlled intercalation contribution, respectively; (**c**) quantitative contribution between capacitive Li^+^ storage and diffusion-controlled intercalation of the hybrid composites.

In engineered nanoscale materials, a considerable amount of Li^+^ can be stored on the surface of active material besides Li^+^ storage via faradaic process. The 3D V_2_O_5_/RGO/CNT architecture develops the mesoporosity and increases the specific surface area dramatically compared to other structures. To investigate the effect of surface area on Li^+^ storage properties in the ternary composite, the intercalation and capacitive contributions to total capacity were characterized from CV curves at various scan rates by using power law relationship^21^,^55^ as the following equation 2 (Fig. 7),


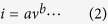


where *i* is current (A), ν is scan rate (mV s^−1^), and a, b are adjustable parameters. A *b*-exponent value is determined from the slope of log(| *i* |) *vs.* log(ν) plot, and the *b*-value of 0.5 indicates the faradaic process such as diffusion-controlled intercalation, conversion, or alloying reactions, while the *b*-value of 1.0 indicates the surface-limited pseudocapacitance^55^. As shown in Fig. 7a, the *b*-value of C2 for 3D V_2_O_5_/RGO/CNT composite is estimated to be 0.53, indicating the diffusion-controlled intercalation, whereas the *b*-values of C1 (0.66) and C3 (0.82) are indicative of the combined behaviors of capacitive and intercalation reactions. In particular, the *b*-values of C1 (0.62) and C3 (0.70) for 2D V_2_O_5_/RGO are lower than those of 3D V_2_O_5_/RGO/CNT, suggesting the more surface Li^+^ storage in the ternary composite. To quantitatively divide the contribution of diffusion-controlled and surface-limited reactions, the following equation 3, describing the current response (*i*) at fixed voltage (V) as the combination of capacitive effect (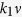) and diffusion-controlled intercalation effect (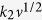), is applied^55^.


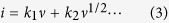


*i* is the current (A) at a given potential, ν is the scan rate (mV s^−1^), and *k*_1_ and *k*_2_ are constants. According to the linear plot of *i*/ν^1/2^ *vs.* ν^1/2^ as shown in Fig. 7b, the electrochemical reactions with phase transitions from the α-phase to the ε-, δ-, and γ-phase (Fig. 6a,b) can be categorized as three regions (C1, C2, and C3, respectively) with the quantitative contribution of intercalation and capacitive reactions as shown in bar graph (Fig. 7c). In both samples, diffusion-controlled intercalation behavior is dominated in the C2 process, and more surface Li^+^ storage in the C1 and C3 than the C2 is observed, which suggests that the faradaic intercalation reaction is developed in the middle of the whole process with a small capacitive effect on total capacity. Moreover, the higher surface Li^+^ storage of 3D V_2_O_5_/RGO/CNT composite is shown in the C1 and C3 processes compared to 2D V_2_O_5_/RGO. It is well known that, for TiO_2_ which is regarded as a typical anode material following the intercalation reaction mechanism, the extent of both domains in the initial solid-solution formation characterized by potential drop and in the further Li^+^ adsorption characterized by final sloping curve is directly proportional to the surface area of material^56^. These two characters correspond to the homogeneous Li^+^ introduction followed by the biphasic transition and the surface Li^+^ storage (i.e. interfacial capacity), respectively^56^. Therefore, the initial potential drop (C1) and the final sloping curve (C3) in the V_2_O_5_-based composites can be correlated proportionally with the surface area, consistent with the higher capacitive effect in the 3D V_2_O_5_/RGO/CNT composite having the higher surface area. Especially for the C3 process, the favorable surface Li^+^ storage in 3D V_2_O_5_/RGO/CNT composite is over diffusion-controlled intercalation, which suggests that the hierarchically porous 3D V_2_O_5_/RGO/CNT architecture can be a rational design to enhance the additional surface Li^+^ storage along with the bulk reaction.

In conclusion, we have successfully prepared a 3D V_2_O_5_/RGO/CNT ternary composite with hierarchical porous structure by using 2D RGO and 1D CNT as conductive network through microwave-assisted hydrothermal method. V_2_O_5_ microsphere assembled by nanoparticles is anchored on the surface of RGO and simultaneously penetrated by CNT. This hierarchical porous structure is designed to develop the surface area and mesoporosity of V_2_O_5_ microsphere, which enhances electrolyte contact and Li^+^ diffusivity. Through *in situ* XRD and *in situ* XANES analyses of the 3D V_2_O_5_/RGO/CNT composite, the phase transformations with structural reversibility upon Li^+^ intercalation/de-intercalation and the electronic and local structure reversibility around vanadium atom during the first cycle are investigated, respectively. Compared to 2D V_2_O_5_/RGO as a control group, the 3D V_2_O_5_/RGO/CNT ternary composite delivers 80 mAh g^−1^ higher capacity at 1 C after 80 cycles and 32 mAh g^−1^ higher capacity at a considerably high rate of 20 C, showing an excellent cycle performance (220 mAh g^−1^ at 1 C) and a superior rate capability (100 mAh g^−1^ at 20 C), respectively. Moreover, favorable Li^+^ diffusivity and enhanced surface Li^+^ storage over diffusion-controlled intercalation reaction are observed in 3D V_2_O_5_/RGO/CNT with the advantage of porous structure, which enables the structure of 3D V_2_O_5_/RGO/CNT ternary composite to become a promising cathode design for high performance LIBs.

## Methods

### Synthesis of graphene oxide nanosheet and functionalized carbon nanotube

GO nanosheets were synthesized from graphite flakes using modified Hummer’s method^57^. Commercially available natural graphite powder and NaNO_3_ were added into 50 ml of concentrated H_2_SO_4_ (sulfuric acid, 98%), and then stirred in ice bath for 30 min. After that, 7 g of KMnO_4_ (potassium permanganate) was added by continuous stirring and 100 ml of deionized (DI) water was slowly poured into the reaction mixture. After dilution with DI water (300 ml), H_2_O_2_ (hydrogen peroxide, 30%) was added and the mixture was centrifuged at 4000 rpm. The precipitate was washed using 5% HCl (hydrochloric acid) to remove residual ions, washed further with DI water to reach the neutral pH, and then dried at 70 °C overnight. To functionalize CNT (oxidized CNT, o-CNT), 0.5 g of commercial multiwalled carbon nanotubes were treated with 50 ml of concentrated HNO_3_ (nitric acid, 65 wt %) under refluxing at 70 °C for 48 h^58^. The mixture was cooled to room temperature, washed and filtered several times using DI water, and then dried in oven at 100 °C to get the functionalized CNT. All chemicals were purchased by Aldrich.

### Synthesis of 3D V_2_O_5_/RGO/CNT ternary composite

The 3D V_2_O_5_/RGO/CNT composite was synthesized by the MAH method, followed by oxidation of V_x_O_y_/RGO/CNT precursor. A homogeneous dispersion of GO (80 mg) and o-CNT (40 mg) in DI water (120 mL) was formed by ultrasonication, and 1 g of V_2_O_5_ powder precursor (purchased from Junsei) was dispersed in the above mixture by magnetic stirring. In addition, the ratio of RGO to CNT was controlled for 2:1 and 1:2 at 80 mg of GO with 40 mg of o-CNT and 40 mg of GO with 80 mg of o-CNT, respectively. An equal molar ratio of ascorbic acid (C_6_H_8_O_6_) was added to the mixture as a reducing agent, and adjusted to pH 1 using 1 M HCl. The above mixture was transferred into Teflon-vessels and treated in MAH reactor (MARS, CEM Corp.) at 200 °C under 300 torr for 30 min. In the MAH process, reduction of GO to graphene as well as formation of 3D V_x_O_y_/RGO/CNT ternary microsphere were achieved simultaneously, and then, the mixture was filtered, washed, and dried at 60 °C overnight in electric oven. The final 3D V_2_O_5_/RGO/CNT ternary composite was obtained by oxidation of as-prepared V_x_O_y_/RGO/CNT composite at 300 °C for 2 h in air.

To investigate the individual role of RGO and CNT in the formation of self-assembled microsphere, V_2_O_5_ with only RGO (120 mg of GO, denoted as only V_2_O_5_/RGO), V_2_O_5_ with double amount of RGO (240 mg of GO, denoted as 2D V_2_O_5_/RGO, The reasons for this condition and notation are explained in the result section), and V_2_O_5_ with only CNT (120 mg of o-CNT, denoted as only V_2_O_5_/CNT) were prepared under the same synthesis process.

### Materials characterization

X-ray diffraction (XRD) patterns were recorded in Bruker D2 PHASER diffractometer with Cu Kα_1_ radiation (λ=1.54056 Å) in the range of 10–80°. The morphologies of sample were characterized by using field-emission scanning electron microscopy (FE-SEM, JEOL JSM7000F) and high-resolution transmission electron microscopy (HRTEM, JEOL JEM2100F) coupled with energy dispersive spectrometer (EDS). The carbon content of sample was measured through thermogravimetric analysis (TGA) by a SEICO INST (TG/DTA 7300) at a heating rate of 10 °C min^−1^ under air flow. X-ray photoelectron spectrometer (XPS) measurement was carried out using VG microtech (ESCA, 2000) with a monochromatic Al Kα source (1486.6 eV), and the results were calibrated by referencing C1s at 284.6 eV. The surface area and pore size distribution were obtained using the N_2_ sorption isotherm by using a Brunauer–Emmett–Teller surface area analyzer (BET, Micromeritics ASAP2000) and the Barrett-Joyner-Halenda (BJH) method.

### Electrochemical measurements

Working electrode was prepared by mixing the active material of 3D V_2_O_5_/RGO/CNT, super P as a conducting agent, and polyvinylidene fluoride (PVDF) as a binder in N-methyl-2-pyrrolidone (NMP) solvent at a weight ratio of 70:15:15. The mixed slurry was uniformly coated on Al foil current collector, and the electrodes were dried at 120 °C overnight. A CR2032 type coin cell, consisting of the 3D V_2_O_5_/RGO/CNT composite as the working electrode and Li metal foil as a counter and a reference electrode, was assembled in a glove box under Ar atmosphere. A Celgard 2300 membrane was used as a separator, and for an organic electrolyte, 1 M LiPF_6_ was dissolved in a mixture of ethylene carbonate (EC) and diethyl carbonate (DEC) with a volume ratio of 1:1.

The galvanostatic charge/discharge measurements (WBCS3000, Wonatech) were carried out on the coin cells in the voltage window of 4.0-2.0 V (*vs.* Li/Li^+^) under various current densities. Galvanostatic intermittent titration technique (GITT) was performed during first discharge and charge processes at a constant current flux (0.1 C) for an interval of 10 min followed by open-circuit stand of 40 min to attain the cell to its steady state equilibrium voltage (E_s_). Cyclic voltammetry (CV) was measured on Wonatech ZIVE MP2 electrochemical workstation at various scan rates over a voltage range of 4.0-2.0 V (*vs.* Li/Li^+^). All electrochemical tests were conducted at room temperature. The *in situ* XRD patterns were collected at beamline 6D in Pohang Light Source (PLS-II) using a position sensitive detector (PSD) with the wavelength of 0.61992 Å at 10 s of exposure time. For easy comparison, two theta angles of all the XRD patterns have been recalculated and converted to the corresponding angles for λ = 1.54 Å (Cu Kα 1 radiation). The X-ray absorption spectroscopy (XAS) measurements were performed at beamline 8C in PLS-II. The oxidation states corresponding to V K-edge in each sample were investigated using a Si (111) double-crystal monochromator detuned to 70% of its original intensity to eliminate the high order harmonics. The *in situ* X-ray absorption near edge spectroscopy (XANES) spectra were collected in transmission mode at 2.5 GeV of electron energy with stored ring current of 400 mA top-up mode. Reference spectra of V metal were collected simultaneously using vanadium foil.

## Additional Information

**How to cite this article**: Palanisamy, K. *et al*. Porous V_2_O_5_/RGO/CNT hierarchical architecture as a cathode material: Emphasis on the contribution of surface lithium storage. *Sci. Rep.* **6**, 31275; doi: 10.1038/srep31275 (2016).

## References

[b1] Kang B. & Ceder G. Battery materials for ultrafast charging and discharging. Nature 458, 190–193 (2009). 19279634 10.1038/nature07853

[b2] Manthiram A. Materials challenges and opportunities of lithium ion batteries. J. Phys. Chem. Lett. 2, 176–184 (2011).

[b3] Kang K., Meng Y. S., Bréger J., Grey C. P. & Ceder G. Electrodes with high power and high capacity for rechargeable lithium batteries. Science 311, 977–980 (2006). 16484487 10.1126/science.1122152

[b4] Wang J. & Sun X. Understanding and recent development of carbon coating on LiFePO_4_ cathode materials for lithium-ion batteries. Energy Environ. Sci. 5, 5163–5185 (2012).

[b5] Yu H. . Cu doped V_2_O_5_ flowers as cathode material for high-performance lithium ion batteries. Nanoscale 5, 4937–4943 (2013). 23629762 10.1039/c3nr00548h

[b6] Whittingham M. S. Electrical energy storage and intercalation chemistry. Science 192, 1126–1127 (1976). 17748676 10.1126/science.192.4244.1126

[b7] Coustier F., Hill J., Owens B. B., Passerini S. & Smyrl W. H. Doped vanadium oxides as host materials for lithium intercalation. J. Electrochem. Soc. 146, 1355–1360 (1999).

[b8] Zeng L., Zheng C., Deng C., Ding X. & Wei M. MoO_2_-ordered mesoporous carbon nanocomposite as an anode material for lithium-ion batteries. ACS Appl. Mater. & Inter. 5, 2182–2187 (2013). 10.1021/am303286n 23438299

[b9] Sun L. . Sulfur embedded in a mesoporous carbon nanotube network as a binder-free electrode for high-performance lithium–sulfur batteries. ACS Nano 10, 1300–1308 (2016). 26695394 10.1021/acsnano.5b06675

[b10] Huang X., Qi X., Boey F. & Zhang H. Graphene-based composites. Chem. Soc. Rev. 41, 666–686 (2012). 21796314 10.1039/c1cs15078b

[b11] Luo J. . Three-dimensional graphene foam supported Fe_3_O_4_ lithium battery anodes with long cycle life and high rate capability. Nano Lett. 13, 6136–6143 (2013). 24219630 10.1021/nl403461n

[b12] Wang C. . Self-healing chemistry enables the stable operation of silicon microparticle anodes for high-energy lithium-ion batteries. Nat. Chem. 5, 1042–1048 (2013). 24256869 10.1038/nchem.1802

[b13] Ranaei Siadat S. O. Electrosynthesis and electrochemical properties of metal oxide nano wire/P-type conductive polymer composite film. J. Electrochem. Sci. Technol. 6, 81–87 (2015).

[b14] Choi S. H. & Kang Y. C. Uniform decoration of vanadium oxide nanocrystals on reduced graphene-oxide balls by an aerosol process for lithium-ion battery cathode material. Chem. Eur. J. 20, 6294–6299 (2014). 24715540 10.1002/chem.201400134

[b15] Han C. . V_2_O_5_ quantum dots/graphene hybrid nanocomposite with stable cyclability for advanced lithium batteries. Nano Energy 2, 916–922 (2013).

[b16] Liu Q. . Graphene-modified nanostructured vanadium pentoxide hybrids with extraordinary electrochemical performance for Li-ion batteries. Nat. Commun. 6, 7127 (2015). 25600907 10.1038/ncomms7127

[b17] Pham-Cong D. . Cathodic performance of V_2_O_5_ nanowires and reduced graphene oxide composites for lithium ion batteries. Curr. Appl. Phys. 14, 215–221 (2014).

[b18] Cheng J. . Self-assembled V_2_O_5_ nanosheets/reduced graphene oxide hierarchical nanocomposite as a high-performance cathode material for lithium ion batteries. J. Mater. Chem. A 1, 10814–10820 (2013).

[b19] Wu Y., Gao G. & Wu G. Self-assembled three-dimensional hierarchical porous V_2_O_5_/graphene hybrid aerogels for supercapacitors with high energy density and long cycle life. J. Mater. Chem. A 3, 1828–1832 (2015).

[b20] Zhang H. . Bifunctional reduced graphene oxide/V_2_O_5_ composite hydrogel: fabrication, high performance as electromagnetic wave absorbent and supercapacitor. ChemPhysChem 15, 366–373 (2014). 24318771 10.1002/cphc.201300822

[b21] Sathiya M., Prakash A. S., Ramesha K., Tarascon J. M. & Shukla A. K. V_2_O_5_-anchored carbon nanotubes for enhanced electrochemical energy storage. J. Am. Chem. Soc. 133, 16291–16299 (2011). 21888392 10.1021/ja207285b

[b22] Zhang Z., Wang L., Xiao J., Xiao F. & Wang S. One-pot synthesis of three-dimensional graphene/CNTs/SnO_2_ hybrid architectures with enhanced lithium storage properties. ACS Appl. Mater. & Inter. 7, 17963–17968 (2015). 10.1021/acsami.5b04673 26237666

[b23] Chen S., Bao P. & Wang G. Synthesis of Fe_2_O_3_–CNT–graphene hybrid materials with an open three-dimensional nanostructure for high capacity lithium storage. Nano Energy 2, 425–434 (2013).

[b24] Sun T. . Facile and green synthesis of palladium nanoparticles-graphene-carbon nanotube material with high catalytic activity. Sci. Rep. 3, 2527–2533 (2013). 23982312 10.1038/srep02527 PMC3755291

[b25] Lingappan N., Van N. H., Lee S. & Kang D. J. Growth of three dimensional flower-like molybdenum disulfide hierarchical structures on graphene/carbon nanotube network: An advanced heterostructure for energy storage devices. J. Power Sources 280, 39–46 (2015).

[b26] Shen L., Zhang X., Li H., Yuan C. & Cao G. Design and tailoring of a three-dimensional TiO_2_–graphene–carbon nanotube nanocomposite for fast lithium storage. J. Phys. Chem. Lett. 2, 3096–3101 (2011).

[b27] Zhang B., Zheng Q. B., Huang Z. D., Oh S. W. & Kim J. K. SnO_2_–graphene–carbon nanotube mixture for anode material with improved rate capacities. Carbon 49, 4524–4534 (2011).

[b28] Choi A. . Microwave-assisted hydrothermal synthesis of electrochemically active nano-sized Li_2_MnO_3_ dispersed on carbon nanotube network for lithium ion batteries. J. Alloys and Compd. 591, 356–361 (2014).

[b29] Sanlés-Sobrido M., Pérez-Lorenzo M., Rodríguez-González B., Salgueiriño V. & Correa-Duarte M. A. Highly active nanoreactors: nanomaterial encapsulation based on confined catalysis. Angew. Chem. Int. Edit. 51, 3877–3882 (2012). 10.1002/anie.201105283 22307952

[b30] Sing K. S. W. . Reporting physisorption data for gas/solid systems with special reference to the determination of surface area and porosity. Pure Appl. Chem. 57, 603–619 (1985).

[b31] Liu Q. . The Structural evolution of V_2_O_5_ nanocystals during electrochemical cycling studied using in operando synchrotron techniques. Electrochim. Acta 136, 318–322 (2014).

[b32] Palanisamy K., Kim Y., Kim H., Kim J. M. & Yoon W.-S. Self-assembled porous MoO_2_/graphene microspheres towards high performance anodes for lithium ion batteries. J. Power Sources 275, 351–361 (2015).

[b33] Mendialdua J., Casanova R. & Barbaux Y. XPS studies of V_2_O_5_, V_6_O_13_, VO_2_ and V_2_O_3_. J. Electron Spectrosc. Relat. Phenom. 71, 249–261 (1995).

[b34] Zhang W., He W. & Jing X. Preparation of a Stable Graphene Dispersion with High Concentration by Ultrasound. J. Phys. Chem. B 114, 10368–10373 (2010). 20701371 10.1021/jp1037443

[b35] Yamamoto O. . Proceedings of the eighth international meeting on lithium batteries: observation of structure change due to discharge/charge process of V_2_O_5_ prepared by ozone oxidation method, using *in situ* X-ray diffraction technique. J. Power Sources 68, 674–679 (1997).

[b36] Meulenkamp E. A., van Klinken W. & Schlatmann A. R. *In situ* X-ray diffraction of Li intercalation in sol–gel V_2_O_5_ films. Solid State Ionics 126, 235–244 (1999).

[b37] Delmas C., Cognac-Auradou H., Cocciantelli J. M., Ménétrier M. & Doumerc J. P. The Li_*x*_V_2_O_5_ system: An overview of the structure modifications induced by the lithium intercalation. Solid State Ionics 69, 257–264 (1994).

[b38] Baddour-Hadjean R., Marzouk A. & Pereira-Ramos J. P. Structural modifications of Li_*x*_V_2_O_5_ in a composite cathode (0 ≤ x < 2) investigated by Raman microspectrometry. J. Raman Spectrosc. 43, 153–160 (2012).

[b39] Pan J., Zhong L., Li M., Luo Y. & Li G. Microwave-assisted solvothermal synthesis of VO_2_ hollow spheres and their conversion into V_2_O_5_ hollow spheres with improved lithium storage capability. Chem. Eur. J. 22, 1461–1466 (2016). 26749240 10.1002/chem.201504259

[b40] Holland G. P., Huguenin F., Torresi R. M. & Buttry D. A. Comparison of V_2_O_5_ xerogels prepared by the vanadate and alkoxide routes using X-Ray absorption and other methods. J. Electrochem. Soc. 150, A721–A725 (2003).

[b41] Giorgetti M. . *In situ* X‐Ray absorption spectroscopy characterization of V_2_O_5_ xerogel cathodes upon lithium intercalation. J. Electrochem. Soc. 146, 2387–2392 (1999).

[b42] Ali G. . Investigation of the Na intercalation mechanism into nanosized V_2_O_5_/C composite cathode material for Na-ion batteries. ACS Appl. Mater. & Inter. 8, 6032–6039 (2016). 10.1021/acsami.5b11954 26889957

[b43] Avansi W. Jr., Ribeiro C., Leite E. R. & Mastelaro V. R. Vanadium pentoxide nanostructures: an effective control of morphology and crystal structure in hydrothermal conditions. Crystal Growth & Design 9, 3626–3631 (2009).

[b44] Ha S. H., Jeong Y. S. & Lee Y. J. Free standing reduced graphene oxide film cathodes for lithium ion batteries. ACS Appl. Mater. & Inter. 5, 12295–12303 (2013). 10.1021/am4044147 24229056

[b45] Lee S. W. . Self-standing positive electrodes of oxidized few-walled carbon nanotubes for light-weight and high-power lithium batteries. Energy Environ. Sci. 5, 5437–5444 (2012).

[b46] Pan A., Wu H. B., Yu L. & Lou X. W. Template-free synthesis of VO_2_ hollow microspheres with various interiors and their conversion into V_2_O_5_ for lithium-ion batteries. Angew. Chem. Int. Ed. 52, 2226–2230 (2013). 10.1002/anie.201209535 23316009

[b47] Shao J. . Low-Cost Synthesis of Hierarchical V_2_O_5_ microspheres as high-performance cathode for lithium-ion batteries. ACS Appl. Mater. & Inter. 5, 7671–7675 (2013). 10.1021/am401854v 23915302

[b48] Li G. . Synthesis of V_2_O_5_ hierarchical structures for long cycle-life lithium-ion storage. J. Mater. Chem. A 3, 1103–1109 (2015).

[b49] Sun Y. . A composite film of reduced graphene oxide modified vanadium oxide nanoribbons as a free standing cathode material for rechargeable lithium batteries. J. Power Sources 241, 168–172 (2013).

[b50] Liang S. . Template-free synthesis of ultra-large V_2_O_5_ nanosheets with exceptional small thickness for high-performance lithium-ion batteries. Nano Energy 13, 58–66 (2015).

[b51] Mai L. . Cucumber-like V_2_O_5_/poly(3,4-ethylenedioxythiophene) & MnO_2_ nanowires with enhanced electrochemical cyclability. Nano Lett. 13, 740–745 (2013). 23311754 10.1021/nl304434v

[b52] Huguenin F., Girotto E. M., Torresi R. M. & Buttry D. A. Transport properties of V_2_O_5_/polypyrrole nanocomposite prepared by a sol-gel alkoxide route. J. Electroanal. Chem. 536, 37–45 (2002).

[b53] Tan H. T., Rui X., Sun W., Yan Q. & Lim T. M. Vanadium-based nanostructure materials for secondary lithium battery applications. Nanoscale 7, 14595–14607 (2015). 26270235 10.1039/c5nr04126k

[b54] Pan A. . Template free synthesis of LiV_3_O_8_ nanorods as a cathode material for high-rate secondary lithium batteries. J. Mater. Chem. 21, 1153–1161 (2011).

[b55] Kim H. . Sodium storage behavior in natural graphite using ether-based electrolyte systems. Adv. Funct. Mater. 25, 534–541 (2015).

[b56] Froschl T. . High surface area crystalline titanium dioxide: potential and limits in electrochemical energy storage and catalysis. Chem. Soc. Rev. 41, 5313–5360 (2012). 22763865 10.1039/c2cs35013k

[b57] Hummers W. S. & Offeman R. E. Preparation of graphitic oxide. J. Am. Chem. Soc. 80, 1339–1339 (1958).

[b58] Datsyuk V. . Chemical oxidation of multiwalled carbon nanotubes. Carbon 46, 833–840 (2008).

